# AGO61-dependent GlcNAc modification primes the formation of functional glycans on α-dystroglycan

**DOI:** 10.1038/srep03288

**Published:** 2013-11-21

**Authors:** Hirokazu Yagi, Naoki Nakagawa, Takuya Saito, Hiroshi Kiyonari, Takaya Abe, Tatsushi Toda, Sz-Wei Wu, Kay-Hooi Khoo, Shogo Oka, Koichi Kato

**Affiliations:** 1 Graduate School of Pharmaceutical Sciences, Nagoya City University, 3-1 Tanabe-dori, Mizuho-ku, Nagoya 467-8603, Japan; 2 Department of Biological Chemistry, Human Health Sciences, Graduate School of Medicine, Kyoto University, 53 Kawahara-cho, Shogoin, Sakyo-ku, Kyoto 606-8507, Japan; 3 Laboratory for Animal Resources and Genetic Engineering, RIKEN Center for Developmental Biology, 2-2-3 Minatojima Minami, Chuou-ku, Kobe 650-0047, Japan; 4 Division of Neurology/Molecular Brain Science, Kobe University Graduate School of Medicine, 7-5-1 Kusunoki-chou Chuo-ku, Kobe 650-0017, Japan; 5 Institute of Biological Chemistry, Academia Sinica, 128, Academia Road Sec 2, Nankang, Taipei 115, Taiwan; 6 Okazaki Institute for Integrative Bioscience and Institute for Molecular Science, National Institutes of Natural Sciences, 5-1 Higashiyama Myodaiji, Okazaki 444-8787, Japan; 7 These authors contributed equally to this work.

## Abstract

Dystroglycanopathy is a major class of congenital muscular dystrophy that is caused by a deficiency of functional glycans on α-dystroglycan (α-DG) with laminin-binding activity. A product of a recently identified causative gene for dystroglycanopathy, AGO61, acted *in vitro* as a protein *O*-mannose β-1, 4-*N*-acetylglucosaminyltransferase, although it was not functionally characterized. Here we show the phenotypes of AGO61-knockout mice and demonstrate that AGO61 is indispensable for the formation of laminin-binding glycans of α-DG. AGO61-knockout mouse brain exhibited abnormal basal lamina formation and a neuronal migration defect due to a lack of laminin-binding glycans. Furthermore, our results indicate that functional α-DG glycosylation was primed by AGO61-dependent GlcNAc modifications of specific threonine-linked mannosyl moieties of α-DG. These findings provide a key missing link for understanding how the physiologically critical glycan motif is displayed on α-DG and provides new insights on the pathological mechanisms of dystroglycanopathy.

Congenital muscular dystrophies and limb-girdle muscular dystrophies are clinically and genetically heterogeneous degenerative diseases that primarily affect voluntary muscles. Dystroglycanopathy is a group of these diseases associated with brain and eye abnormalities at the severe end of the clinical spectrum, including Walker–Warburg syndrome (WWS), muscle-eye-brain (MEB) diseases, Fukuyama-type congenital muscular dystrophy (FCMD), and congenital muscular dystrophy type 1D (MDC1D). The hallmark of these diseases is hypoglycosylation of α-dystroglycan (α-DG)^1^,^2^, which along with β-DG is cleaved from a precursor protein encoded for by a single gene by post-translational processing^3^. A dystrophin glycoprotein complex that is formed from several intracellular, transmembrane, and extracellular proteins including α- and β-DG subunits connects the cytoskeleton of a muscle fiber to surrounding extracellular matrix components, such as laminin, agrin, and perlecan, depending on the glycosylation status of α-DG^4^,^5^.

To date, 13 genes have been identified that are associated with dystroglycanopathies, among which 8 genes have been characterized as those encoding enzymes responsible for the formation of the functional α-DG glycans. Protein *O*-mannosyltransferase 1 (POMT1)^6^, POMT2^7^,^8^, and protein *O*-mannose β-1,2-*N*-acetylglucosaminyltransferase 1 (POMGnT1)^9^ along with GDP-mannose pyrophosphorylase (GMPPB)^10^ are involved in the biosynthesis of *O*-mannosyl glycans on α-DG. The outer regions of laminin-binding glycans consist of Xyl-GlcA repeat sequences, the formation of which is catalyzed by like-acetylglucosaminyltransferase (LARGE), a causative gene product for MDC1D^11^,^12^. Furthermore, phosphorylated *O*-mannosylation was identified on recombinant α-DG, the laminin-binding glycans of which were shown to be degraded by HFaq treatment that hydrolyzes phosphoester linkages^13^. Thus, the laminin-binding region is presumably linked to α-DG through post-phosphoryl *O*-manosylation. However, the functionally relevant glycan structure of α-DG has not been well delineated, notwithstanding that several mutants associated with dystroglycanopathy have been identified in fukutin^14^, fukutin-related protein (FKRP)^15^, UDP-GlcNAc:βGal β-1,3-*N*-acetylglucosaminyltransferase 1 (B3GNT1)^16^, isoprenoid synthase domain containing (ISPD)^17^,^18^,^19^, and transmembrane protein 5 (TMEM5)^19^, which are associated with impaired formation of α-DG laminin-binding glycans.

Recently, AGO61 (also known as GTDC2) was newly identified as a causative gene product associated with WWS based on the results of whole-exome sequencing, homozygosity mapping, and morpholino-mediated knockdown of an AGO61 zebrafish ortholog^20^. It was subsequently characterized as a protein *O*-mannose β-1,4-*N*-acetylglucosaminyltransferase (POMGnT2) based on its *in vitro* enzymatic activities against a synthetic peptide substrate carrying a single *O*-Man^21^. Although a further β3GalNAc-extended and phosphorylated GlcNAcβ1-4Man-*O*- unit by β-1,3-*N*-acetylgalactosaminyltransferase 2 (B3GALNT2)^21^,^22^ and protein kinase-like protein SGK196^21^,^23^, respectively, was inferred to be essential for the formation of functional laminin binding glycans on αDG, the precise roles of AGO61 *in vivo* remain to be determined. Here, we characterized the phenotypes of AGO61-knockout mice and determined that AGO61 mediated the formation of laminin-binding glycans on α-DG.

## Results

### AGO61-knockout mice exhibit abnormal neuronal migration

**Figure 1:**
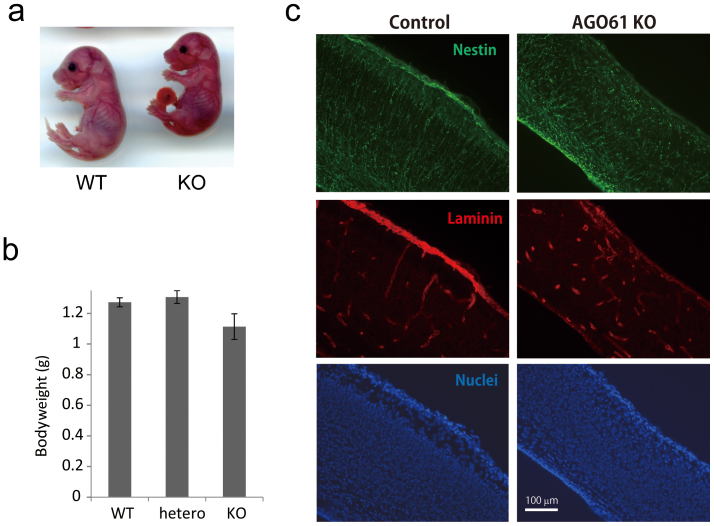
AGO61-KO mice exhibit neuron migration defects. (a) Phenotypes of AGO61 KO and WT pups at embryonic day 17.5. (b) Body weights of wild type (WT), heterozygous (hetero), and KO pups at postnatal day 0 (n = 4–6 for each genotype). Results are means ± SDs. (c) Brain sagittal sections from AGO61-KO and WT pups at embryonic day 17.5 were stained with anti-nestin and anti-laminin antibodies used as primary antibodies, and then with Alexa Fluor 488-conjugated anti-rat IgG (*green*) and Alexa Fluor 595-conjugated anti-rabbit IgG (*red*) used as secondary antibodies, respectively. Nuclei were stained with DAPI (*blue*).

We subjected the mouse AGO61 locus to targeted disruption. A null allele was generated by replacing the first coding exon with a neomycin resistance gene (Supplementary Fig. S1a). Mice that were heterozygous for the AGO61 mutation appeared grossly normal and were fertile. The progeny of a heterozygous intercross had an approximately 1:2:1 ratio of wild type and heterozygous AGO61, and a homozygous AGO61 ratio that was indicative of Mendelian inheritance. However, the newborns of homozygotes were slightly smaller than the other genotypes and died within the first day of birth (Fig. 1a and b).

AGO61 is mainly expressed in the central nervous system^20^. AGO61-knockout mouse brains exhibited abnormal basal lamina formation and the radial glia endfoot had detached from the basal membrane (Fig. 1c). Moreover, nuclear staining revealed defects in neuronal migration and laminar organization in the AGO61-KO mouse cerebral cortex (Fig. 1c). These neurodevelopmental abnormalities are commonly seen in dystroglycanopathy mouse models^24^,^25^,^26^, which suggested an essential role for AGO61 in the functional maturation of α-DG *in vivo*.

### AGO61 is indispensable for the formation of laminin-binding glycans of α-DG

**Figure 2:**
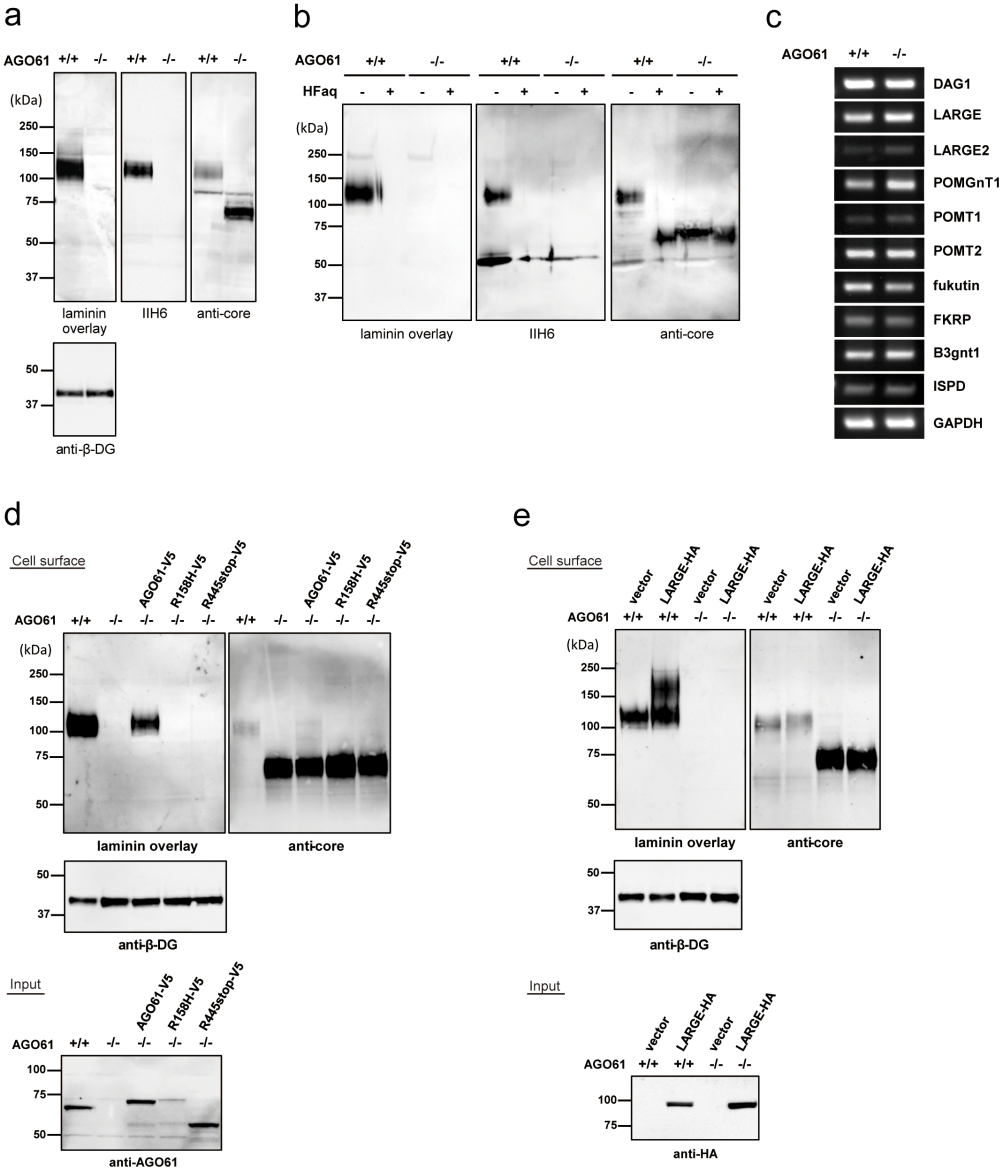
AGO61 is indispensable for the formation of laminin-binding glycans of α-DG. (a) WGA-enriched brain lysates prepared from WT and AGO61-KO pups at embryonic day 17.5 were subjected to laminin overlay and immunoblot analysis using IIH6, anti α-DG core, and anti-β-DG antibodies. The full-length blot with anti-β-DG antibody is presented in Supplementary Fig. S7a. (b) Chemical dephosphorylation of α-DG from WGA enriched brain lysates. Brain lysates were treated with HFaq and then analyzed by laminin overlay and Western blot using IIH6 and anti α-DG core antibodies. (c) mRNA expression of DAG1, LARGE, LARGE2, POMGnT1, POMT1, POMT2, fukutin, FKRP, B3GNT1, ISPD, and GAPDH in brain tissue from WT and AGO61 KO pups at embryonic day 17.5 were analyzed by RT-PCR. GAPDH was used as an internal control. (d) AGO61 and its mutants with loss-of-function mutations were transfected into *AGO61*-deficient MEFs. Cell surface proteins were biotinylated, pull down, and analyzed by laminin overlay and Western blot with anti α-DG core and β-DG antibodies. Cell lysates were also analyzed for AGO61 expression by Western blot using an anti-AGO61 antibody. The full-length blots with anti-β-DG and anti-AGO61 antibodies are presented in Supplementary Figs. S7b and S7c, respectively. (e) LARGE was transfected into control (+/+) or AGO61-deficient (−/−) MEFs. Cell surface proteins were biotinylated, pulled down, and analyzed by laminin overlay and Western blot using anti α-DG core and β-DG antibodies. Cell lysates were analyzed for LARGE-HA expression by Western blot using an anti-HA antibody. The full-length blots with anti-β-DG and anti-HA antibodies are presented in Supplementary Figs. S7d and S7e, respectively.

For biochemical analysis, we enriched DG from mouse embryonic brains (embryonic day 17.5) with wheat germ agglutinin (WGA) beads and then performed laminin overlay and Western blot analyses using IIH6, which recognizes laminin-binding glycans on α-DG, and anti-α-DG core antibodies. AGO61-KO embryonic mouse brains exhibited α-DG hypoglycosylation, which indicated a lack of laminin-binding glycans (Fig. 2a). An immunoreactive band of an α-DG core of AGO61-KO mice migrated to a position similar to that of control mouse α-DG treated with HFaq (Fig. 2b). Furthermore, there were no significant differences between WT and KO mice brains in the expression levels of other dystroglycanopathy-associated genes (Fig. 2c). These results indicated that AGO61 was involved in the formation of laminin-binding glycans on α-DG.

To confirm these findings, we expressed the AGO61 protein in *AGO61*-deficient mouse embryonic fibroblasts (MEFs). AGO61 protein expression rescued the defect in laminin-binding glycans, whereas mutants with alleles associated with WWS (R158H and R445stop)^20^ had no rescue capability (Fig. 2d). Although it has been reported that several dystroglycanopathy models with mutations in POMGnT1, LARGE, or fukutin could be recovered by LARGE overexpression, laminin-binding glycans were not rescued by LARGE overexpression in AGO-deficient MEFs (Fig. 2e). LARGE is located in the Golgi apparatus and is responsible for the formation of the functional Xyl-GlcA repeats of laminin-binding glycans^12^,^27^,^28^, whereas AGO61 was localized in the endoplasmic reticulum (ER) (Supplementary Fig. S2), consistent with the previously reported result obtained with HEK293 cells^21^. These results indicated that AGO61 was actively involved in an early stage of laminin-binding glycan formation.

### AGO61 modifies GlcNAc residues at specific sites on α-DG

**Figure 3:**
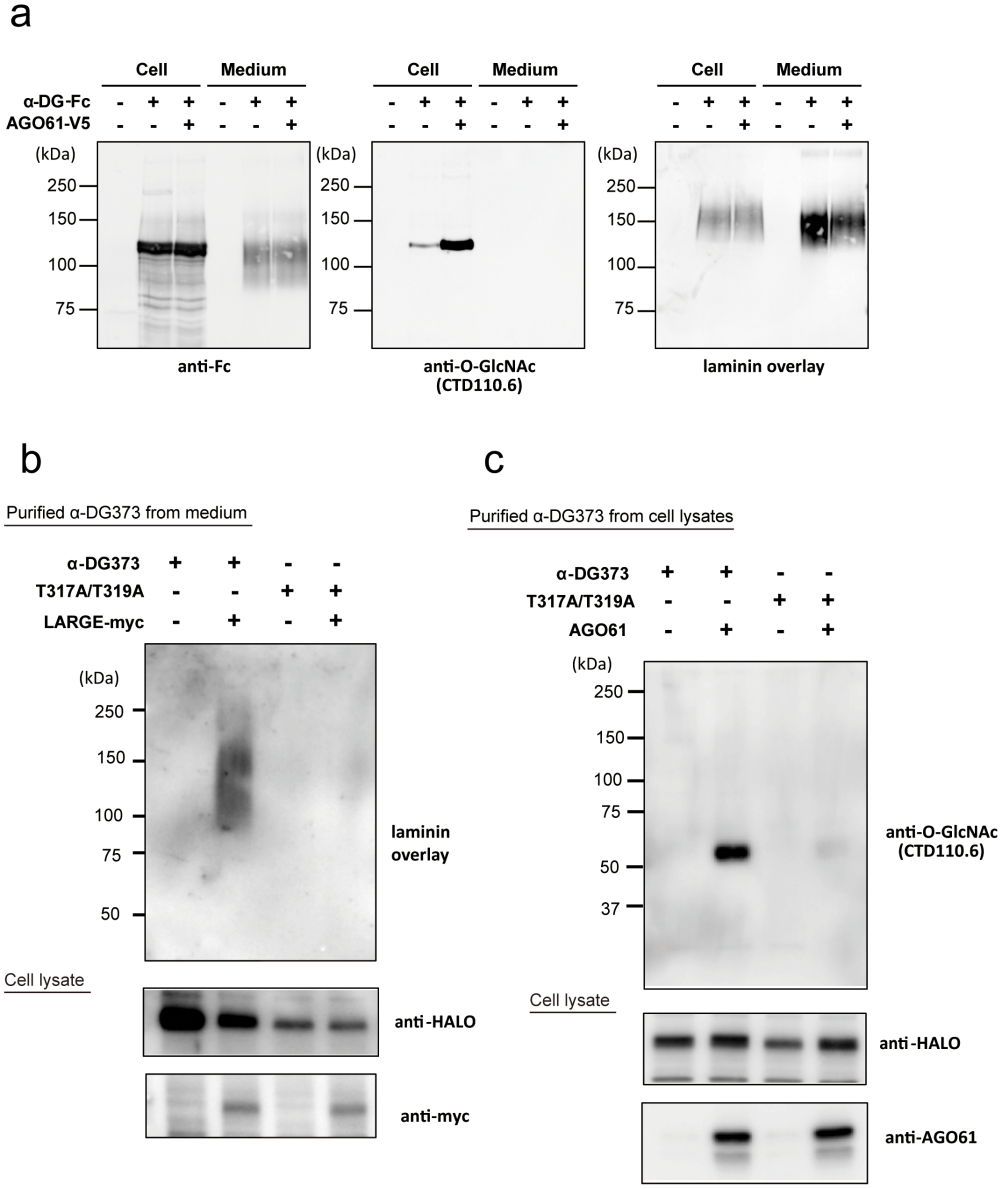
AGO61 modifies GlcNAc residues at specific sites on α-DG. (a) α-DG-Fc was transiently transfected with or without AGO61 into COS1 cells. α-DG-Fc recombinant proteins were collected from cell lysates and culture media using protein A resin and analyzed for laminin overlay and Western blot using anti-Fc and anti-*O*-GlcNAc antibodies. (b) α-DG373-HALO and its mutant T317A/T319A were transiently transfected with or without LARGE-myc into COS7 cells. HALO-fused proteins were collected from medium using HALO resin followed by digestion with TEV protease and then analyzed by laminin overlay. Cell lysates were analyzed for the expression of HALO-fused proteins and LARGE-myc by Western blot using anti-HALO and anti-myc antibodies. The full-length blots with anti-HALO and anti-myc antibodies are presented in Supplementary Figs. S7f and S7g, respectively. (c) α-DG373-HALO and its mutant T317A/T319A were transiently transfected with or without AOG61 into COS7 cells. HALO-fused proteins were collected from the cell lysates using HALO resin followed by digestion with TEV protease and then analyzed by Western blot using an anti-*O*-GlcNAc antibody (CTD110.6). The cell lysates were analyzed for the expression of HALO-fused proteins and AGO61 by Western blot using anti-HALO and anti-AGO61 antibodies. The full-length blots with anti-HALO and anti-AGO61 antibodies are presented in Supplementary Figs. S7h and S7i, respectively.

Most recently, it was reported that AGO61 had POMGnT activity, which contributed to the formation of a previously identified phosphorylated *O*-mannosyl trisaccharide [GalNAc-β3-GlcNAc-β4-(phosphate-6-)Man]^21^. However, AGO61 belongs to the GT61 family, which includes the recently identified extracellular protein *O*-β-*N*-acetylglucosaminyltransferase. To detect all likely GlcNAc modifications by AGO61, we used an anti-*O*-GlcNAc antibody (clone: CTD110.6) with known wide cross-reactivity, including reactivity against terminal GlcNAc-β1-4GlcNAc^29^ (Fig. 3a). AGO61-dependent GlcNAc modification was detected by CTD110.6 but not by another anti-*O*-GlcNAc antibody (HGAC85) (Supplementary Fig. S3). Interestingly, AGO61-dependent GlcNAc modifications were detected in α-DG-Fc prepared from cell lysates but not in those secreted into the medium irrespective of HFaq treatment (Fig. 3a and Supplementary Fig. S4).

To identify the AGO61-dependent GlcNAc modification sites on α-DG, we generated 3 types of α-DG deletion mutants with C-termimal HALO tags (Supplementary Fig. S5). AGO61-dependent GlcNAc modifications were detected in all these mutants, which indicated that the modification had occurred in the N-terminal 62-residue segment of the mucin-like domain. This domain contains the previously identified sites (Thr-317 and Thr-319) that display the laminin-binding glycans produced by LARGE^30^. Using α-DG373 and its T317A/T319A mutant, we confirmed that these threonine residues displayed laminin-binding glycans (Fig. 3b). Interestingly, the T317A/T319A mutant of α-DG373 exhibited little GlcNAc modification despite AGO61 expression (Fig. 3c).

**Figure 4:**
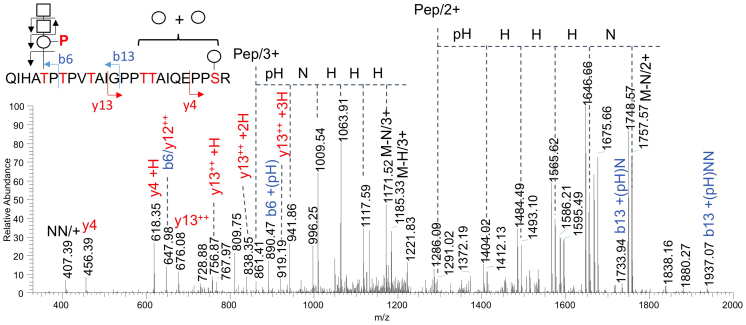
Determination of the presence of a phosphorylated trisaccharide on T317 of α-DG428 co-expressed with AGO61 in COS7 cells. α-DG428 was purified using a HaloTag protein purification system from cell lysates co-transfected with α-DG428-HALO and AGO61, separated by SDS-PAGE, and in-gel digested with trypsin (Supplementary Fig. S6). The extracted peptides/glycopeptides were directly analyzed by LC-MS/MS. The successive neutral losses of HexNAc and PHex from both the doubly and triply charged molecular ions overlapped with those of the additional Hex and collectively defined the m/z of the bare peptide core with the fitted P_1_Hex_4_HexNAc_2_ glycosyl composition. The y4 + Hex and y13 + Hex_1-3_ fragment ions localized the additional 3 Hex on the C-terminal half of the peptide, whereas the b13 + PHex + HexNAc_1-2_ ions established the HexNAc_2_PHex substituent on the N-terminal half along with the b6 + PHex ion that further identified it on T317, as annotated. Key: Pep, peptide core; circle and H, Hex; square and N, HexNAc; P, phospho-; pH, phosphorylated Hex; M, molecular ion.

Moreover, by LC-MS/MS analysis of the tryptic glycopeptides derived from recombinant α-DG428 expressed in the presence or absence of AGO61 in COS7 cells, among other glycoforms, we detected one that carried three Hex, two HexNAc, and a phosphorylated Hex (PHex) on QIHA^317^TP^319^TPVTAIGPPTTAIQEPPSR (Fig. 4). In accordance with the substrate specificity of SGK196, which phosphorylates the trisaccharide GalNAcβ-3GlcNAcβ-4Man but not the single Man^21^, the detected b6 + PHex ion localized the phosphorylated trisaccharide at T317. This was corroborated by the b13 ions that carried an intact HexNAc_2_PHex moiety and Hex-containing y4 and y13 ions, which localized the additional *O*-Hex substituents at the C-terminal half distal from T317.

### AGO61 is a priming enzyme for the formation of laminin-binding glycans

**Figure 5:**
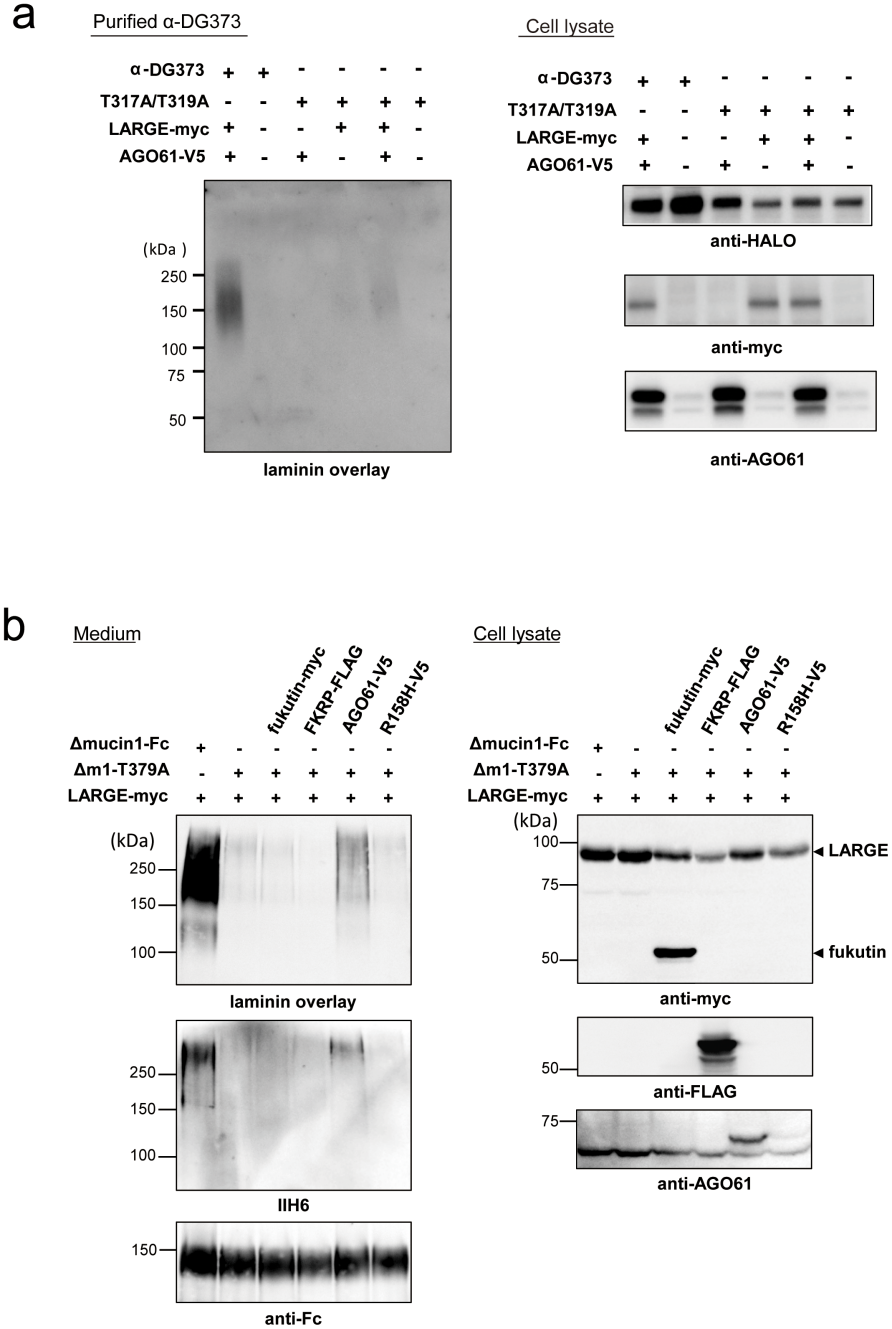
Laminin-binding glycans are primed by an AGO61-dependent GlcNAc modification. (a) α-DG373-HALO and its mutant T317A/T319A were transiently transfected with or without AOG61 and LARGE-myc into COS7 cells. HALO-fused proteins were collected using HALO resin followed by digestion with TEV protease and then analyzed by laminin overlay. Cell lysates were analyzed for the expression of HALO-fused proteins, LARGE-myc, and AGO61 by Western blot using anti-HALO, anti-myc, and anti-AGO61 antibodies. The full-length blots with anti-HALO, anti-myc, anti-AGO61 antibodies are presented in Supplementary Figs. S7j, S7k, and S7l, respectively. (b) Δmucin1-Fc and Δm1-T379A were transiently transfected with or without fukutin-myc, FKRP-FLAG, AGO61-V5, and AGO61-R158H-mutant-V5 (R158H-V5) into LARGE overexpressing COS1 cells. Secreted proteins were pulled down from the culture medium and analyzed by laminin overlay assay and Western blot using IIH6 and anti-Fc antibodies (medium). Anti-Fc antibody was used to monitor protein loading. Cell lysates were analyzed for the expression of LARGE-myc, fukutin-myc, FKRP-FLAG, and AGO61-V5 by Western blot using anti-myc, anti-FLAG, and anti-AGO61 antibodies. The full-length blots with anti-Fc, anti-FLAG and anti-AGO61 antibodies are presented in Supplementary Figs. S7m, S7n and S7o, respectively.

LARGE-dependent hyperglycosylation was undetectable for the T317A/T319A mutant of α-DG373, whereas the formation of laminin-binding glycans was slightly enhanced when LARGE was co-overexpressed with AGO61 (Fig. 5a). This indicated that an alternate *O*-Man residue(s) within α-DG373 (other than T317 and T319) was utilized as the LARGE-dependent modification site by the action of AGO61. Based on this result, we hypothesized that AGO61 was a key regulator for the expression of a laminin-binding glycan on a specific *O*-Man residue. To test this, we used Δmucin1-Fc, an α-DG-Fc mutant with only one major LARGE-dependent modification site, Thr-379, as it lacked Arg311 through Ile370^31^. As expected, a T379A mutant of Δmucin1-Fc (Δm1-T379A) barely exhibited any laminin-binding activity even under LARGE-overexpression conditions (Fig. 5b). However, LARGE and AGO61 co-overexpression resulted in the production of an IIH6-positive laminin-binding glycan on Δm1-T379A. Moreover, this effect was not observed with an AGO61 mutant (R158H) and several other dystroglycanopathy-associated gene products, including fukutin and FKRP. This supported our hypothesis that AGO61 was the priming enzyme required for determining candidate sites where a laminin-binding glycan was formed.

## Discussion

AGO61-KO mice lacked laminin-binding glycans and exhibited phenotypes similar to those of known dystroglycanopathy mutants, as reflected by abnormalities in neural migration and basal lamina formation (Figs. 1 and 2). These defects were also observed in dystroglycanopathy mouse models with mutations of DG, fukutin, B3GNT1, or ISPD, due to a lack of laminin-binding glycans displayed on α-DG^24^,^25^,^26^. Our results show, firstly, that AGO61 is indispensable for the formation of laminin-binding glycans.

Although it has been shown that the laminin-binding glycans of α-DG are extended through a phosphodiester linkage and phosphorylated *O*-Man was identified on recombinant α-DG, their entire structures from the innermost *O*-Man to the outer post-phosphoryl moiety have not been established. It was suggested that the formation of a phosphorylated *O*-mannosyl trisaccharide, GalNAc-β3-GlcNAc-β4-(phosphate-6-)Man, as catalyzed by AGO61, B3GALNT2, and SGK196^21^, served as the base for the extension of laminin-binding glycans. More specifically, AGO61 can attach a β-GlcNAc moiety at the 4-position of α-DG-*O*-mannose, which is followed by β3-GalNAc attachment by B3GALNT2 and subsequently SGK196-catalyzed mannose phosphorylation. Our LC-MS/MS data provide the first evidence for the implicated presence of this phosphorylated trisaccharide structure on Thr-317 in the mucin-like region of α-DG, which was previously identified as the formation site of laminin-binding glycans (Fig. 4). In the present study, AGO61-dependent GlcNAc modifications were detected on α-DG-Fc prepared from cell lysates but not in those secreted into the medium irrespective of HFaq treatment (Fig. 3a and Supplementary Fig. S4). These results suggested that additional GalNAc-modifications masked the GlcNAc residues during secretion. The elongation of laminin-binding glycans based on a phosphorylated *O*-mannosyl trisaccharide may be mediated by some uncharacterized glycosyltrasferases encoded for by some dystroglycanopathy-associated genes.

While the mucin-like domain of α-DG possesses numerous serine/threonine residues as potential *O*-mannosylation sites^32^,^33^,^34^,^35^, AGO61 promotes the GlcNAc modification of *O*-Man at specific sites, as best exemplified by Thr-317 and Thr-319, for the formation of laminin-binding glycans (Fig. 4). Furthermore, in α-DGs mutated at these sites (T317A/T319A-α-DG373 and T379A-Δmucin1-Fc), laminin-binding activity was lost even when LARGE was overexpressed, although it could be rescued solely by overexpressing AGO61. These results indicated that functional α-DG glycosylation was primed by an AGO61-dependent GlcNAc modification.

In summary, we demonstrated that AGO61 served as an essential POMGnT by priming the formation of laminin-binding glycans on α-DG. Our findings provide a critical missing link for understanding the laminin-binding glycan structures displayed on α-DG and provide therapeutic insights for dystroglycanopathy.

## Methods

### cDNA construction

Expression plasmids for human IgG-Fc fused α-DG (α-DG-Fc) and mouse LARGE fused with myc epitope were constructed as described previously^36^. For the expression plasmid for mouse LARGE fused with HA tag, the cording sequence of mouse LARGE was amplified by PCR and cloned into phCMV3 (Genlantis). For an AGO61 expression vector, the encoding sequence of mouse AGO61 was amplified by PCR and cloned into pcDNA3.1/V5-His-TOPO (C-terminal hexahistidine and V5 tags) (Invitrogen). Amino acid substitutions and deletion mutants of α-DG (α-DG373, α-DG428, and α-DG485) were made using standard PCR and genetic engineering techniques. These mutants were cloned into pFC14K HaloTag CMV Flexi vectors (C-terminal HALO tags) (Promega). For fukutin and FKRP expression vectors, the encoding sequences of mouse fukutin and FKRP were amplified by PCR and cloned into C terminal p3XFLAG-CMV (sigma) and pSecTag2 (Invitrogen), respectively.

### Antibodies

We used the following primary antibodies in this study: anti-AGO61 monoclonal antibody (mAb) (Atlas Antibodies AB); anti-*O*-GlcNAc (CTD110.6) mAb (Cell Signaling Technology); anti-*O*-GlcNAc (HGAC85) mAb (Novus Biologicals); anti-Halo mAb (Promega); anti-laminin polyclonal antibody (pAb) (Sigma); anti-Myc mAb and IIH6 mAb (Millipore); anti-HA mAb (Nakalai Tesque); anti-Fc pAb (Jackson ImmunoResearch); anti-β-DG mAb (Novocastra); and α-DG core pAb (goat polyclonal antibody against the C-terminal domain if the a-DG polypeptide)^27^. The following secondary antibodies were used for Western blot analysis: horseradish peroxidase (HRP)-conjugated anti-rabbit IgG mAb (Invitrogen); HRP-conjugated anti-mouse IgM mAb (Thermo Scientific); and HRP-conjugated anti-mouse IgG mAb (Invitrogen).

### Generation of AGO61 mutant mice

AGO61 mutants (Acc. No. CDB0628K: http://www.cdb.riken.jp/arg/mutant%20mice%20list.html) were generated as described at http://www.cdb.riken.jp/arg/Methods.html (Fig. S1 in Supporting Information). The described genotypes were consistently observed irrespective of their ES cell clone of origin, number of successive brother–sister mating generations, or backcrossing with C57BL/6J mice (more than 10 generations). PCR genotyping of AGO61 alleles was performed using Ex Taq polymerase (Takara) and the following primers as indicated in Fig. S2 (Supporting Information): an AGO61-specific forward primer (AgoF 5′-GTTGGTGGGCTAGGCAGATA-3′); a neo-specific forward primer (NeoF 5′-TCGCCTTCTTGACGAGTTCT -3′); and a common reverse primer (CoR 5′-CCTCCTGGTTGGATTTGAGA-3′).

### Mice

All animal treatments and experiments were done in accordance with the guidelines and regulations of Nagoya City University. The protocol was approved by the Committee for Animal Experiments of the Graduate School of Pharmaceutical Sciences, Nagoya City University.

### Cell culture and transfection

Neuro2a, COS1, and COS7 cells were maintained in Dulbecco's modified Eagle's medium (DMEM, Life Technologies) supplemented with 10% fetal bovine serum (FBS) in 5% CO2 at 37°C. For cDNA transfection, cells were grown overnight and transfected using Lipofectamine 2000 (Life Technologies) according to the manufacturer's instructions.

Mouse embryo fibroblasts (MEFs) were prepared from individual AGO61 mutant and wild-type embryos at embryonic day 13.5. An embryo without a head and internal organs was minced and treated with 0.1% trypsin at 37°C for 30 min. These cells were grown and maintained in DMEM containing 10% FBS. Rescue experiments were conducted with AGO KO MEFs using expression vectors for AGO61 and its mutants with loss of function mutations (R158H and R445stop) using an NEPA21 electroporator (NEPA Gene).

### Purification of HALO-tag fused proteins

Recombinant α-DG mutants were purified using HaloLink resin (Promega). A HALO-tag was removed by proteolytic cleavage using HaloTEV protease (Promega) according to the manufacturer's instructions. Purified proteins were separated by SDS-PAGE and subjected to silver staining or Western blotting.

### Western blotting and laminin overlay

Western blotting and laminin overlay were performed as described previously^36^,^37^.

### HFaq treatment

To hydrolyze phosphoester linkages, lysates of brain tissues were treated with ice-cold 48% HFaq (WAKO) at 0°C for 16 h. After removing HFaq with N_2_ gas, the resulting lysates were subjected to SDS-PAGE followed by Western blot analysis. After SDS-PAGE, proteins were transferred onto PVDF membranes. The membranes were incubated with ice-cold 48% HFaq at 4°C for 16 h. Control samples were prepared similarly and treated with ice-cold water. The membranes were then washed thrice with ice-cold water to remove residual HF and subjected to laminin overlay assay or immunoblotting.

### Glycosidase treatments

Recombinant α-DG was treated with β-*N*-acetylhexosaminidase (New England Biolabs), β-*N*-acetylglucosaminidase (New England Biolabs), or α-*N*-acetylgalactosaminidase (New England Biolabs) according to the manufacturer's instructions. These specimens were subjected to Western blot analysis with an anti-*O*-GlcNAc antibody, CTD110.6.

### LC-MS/MS and data analysis

In-gel tryptic digestion of recombinant proteins was performed as previously described^37^. Extracted peptides were solubilized in 0.1% formic acid and analyzed by nanospray LC-MS2 using a nanoACQUITY UPLC System (Waters, Milford, MA, USA) coupled to an LTQ-Orbitrap Velos (Thermo Scientific) through a PicoView (PV550, New Objective, Woburn, MA, USA) nanospray interface. Peptide mixtures were loaded onto a 75 μm × 250 mm nanoACQUITY UPLC BEH130 column packed with C18 resin (Waters, Milford USA) and separated at a flow rate of 300 nl/min using a linear gradient of 5%–50% of solvent B (95% acetonitrile with 0.1% formic acid) in 80 min, followed by a sharp increase to 85% B in 1 min and held at 85% B for another 10 min. Solvent A was 0.1% formic acid in water. The data acquisition cycle included a full MS scan (m/z 400–2000) recorded in the Orbitrap analyzer at 30,000 resolution, followed by data dependent MS2 acquisition of the 20 most intense peptide ions in the linear ion trap. Precursor ion isolation width was set at 3 Th and all singly charged precursors were excluded. The automatic gain control (AGC) targets for Orbitrap full MS and ion trap MSn were set at 1 × 106 and 1 × 104, respectively. All MS/MS raw data were processed using DeconMSn version 2.2.2.2 and directly searched against the α-DG protein sequence using the Mascot Daemon 2.4 server with the following criteria: trypsin digestion; fixed modification set as carbamidomethyl (Cys); variable modifications set as oxidation (Met), Hex (Thr/Ser), HexNAc (Thr/Ser), and PhophoHex (Thr/Ser); up to one missed cleavage allowed; and mass accuracy of 10 ppm for the parent ion and 0.60 Da for fragment ions. Closely eluting glycopeptides corresponding to other glycoforms were further identified manually by inspecting the raw data.

### Semi-quantitative RT-PCR

Semi-quantitative RT-PCR was done as described previously^36^. In brief, total RNA was isolated from embryonic brains at embryonic day 17.5 carrying wild-type and mutant genotypes using TRIzol reagent (Life Technologies). cDNAs were synthesized from the total RNAs as templates using SuperScriptIII reverse transcriptase (Life Technologies). To determine mRNA expression levels, PCR was done with the following primer pairs: DAG1: GCCAGATTCGCCCAACACTGACAAT and CCACCCAGGCATCTACCCTGTCAAT; LARGE: GTCAGATGCAGAAGCCCAGCAGTTC and TGGGGAAAGAGAGTCTGTAGCGCAG; LARGE2: CGAGAGCTGCTCACTCTGAT and GGCATCCAAAGAGCTCTCTT; POMGnT1: TCGTGGGACGAAAAGGAGGTCC and TGGGCCGGTTCCCTGCAATG; POMT1: TTGCCCGCATCACCCAAGGC and GGCTGCGACATCGTGCGTGTT; POMT2: TTGCTGGCTACCTGAGCGGG and AGGGGGCAGAGAAAGGCCTGTT; fukutin: CACTATTTGTCTGCAAGGAATGGAC and CTTGCTTTCAGTCTTTAGGCATTGA; FKRP: CTTCTGTCCCGCTTCAGTTC and AACCAGAGAGAGCCCAGTCA; B3gnt1: AATCAGCCAGGCTTGTGAGC and TGGAGGCATGTTTCTTACCCC; ISPD: TGGATCACATAGGCGGAGAC and GCTTCTGCTCATCCTGTGAGT; and GAPDH: GGAAGGGCTCATGACCACAGTCCAT and CATACTTGGCAGGTTTCTCCAGGCG. PCR products were analyzed by agarose gel electrophoresis using 1% agarose gels.

### Immunohistochemistry

Mouse embryonic brains at embryonic day 17.5 carrying wild-type and mutated genotypes were fixed in phosphate-buffered saline containing 4% paraformaldehyde, embedded in O.C.T. compound (Sakura Finetechnical, Tokyo, Japan), and frozen in liquid nitrogen. Cryosections (20 μm thick) were prepared from the embedded brains and stained with anti-nestin and anti-laminin antibodies. For a secondary antibody, Alexa Fluor 488-conjugated anti-goat IgG antibody (Life Technologies) or Alexa Fluor 546-conjugated anti-rabbit IgG antibody (Life Technologies) was used. Nuclei were stained with DAPI (1 μg/mL; Sigma-Aldrich). Stained sections were photographed under a Nikon Eclipse TE300 fluorescent microscope (Nikon).

### Immunocytochemistry

Neuro2a cells transfected with an AGO expression vector and subcellular localization vectors (pDS-red2-ER or pAcGFP-Golgi; Clontech, Palo Alto, CA) were plated onto chamber slides (Nalge Nunc International) and fixed in PBS containing 4% paraformaldehyde. Cells were treated for 2 h with PBS containing 3% fetal bovine serum and 0.1% Triton X-100, and then stained with primary antibodies, including an anti-AGO61 monoclonal antibody (BD Biosciences, San Jose, CA), and a secondary antibody, an anti-rabbit IgG antibody conjugated with Alexa Fluor 488 or Alexa Fluor 595 (BD Biosciences). Nuclei were stained with 2 μg/mL of Hoechst 33258 (Sigma-Aldrich). Stained cells were photographed under a Nikon Eclipse TE300 fluorescent microscope.

## Author Contributions

H.Y., S.O., T.T. and K.Kato designed the research; H.K. and T.A. established AGO null mice; H.Y., N.N. and T.S. performed the biochemical exprerimets; H.Y., S.W. and K. Khoo performed LC-MS/MS analyses; H.Y., S.O., K. Khoo and K. Kato contributed to the conception of this work and wrote the paper.

## Supplementary Material

Supplementary Information Supplementary Figures
