# Supplementary material for: AGO61-dependent GlcNAc modification primes the formation of functional glycans on α-dystroglycan
Source: Sci Rep. 2013 Nov 21;3:3288. doi: 10.1038/srep03288 (PMC3836086; doi:10.1038/srep03288)
Supplement: Supplementary Information — Supplementary Figures [file srep03288-s1.pdf]

Supplementary information

AGO61-dependent GlcNAc modification primes the formation of functional glycans on α-dystroglycan

Hirokazu Yagi[1], Naoki Nakagawa[2], Takuya Saito[1], Hiroshi Kiyonari[3], Takaya Abe[3], Tatsushi Toda[4], Sz-Wei Wu[5], Kay-Hooi Khoo[5], Shogo Oka[2], and Koichi Kato[1,6]

[1]Graduate School of Pharmaceutical Sciences, Nagoya City University, 3-1 Tanabe-dori, Mizuho-ku, Nagoya 467-8603, Japan; [2]Department of Biological Chemistry, Human Health Sciences, Graduate School of Medicine, Kyoto University, 53 Kawahara-cho, Shogoin, Sakyo-ku, Kyoto 606-8507, Japan; [3]Laboratory for Animal Resources and Genetic Engineering, RIKEN Center for Developmental Biology, 2-2-3 Minatojima Minami,Chuou-ku, Kobe 650-0047, Japan; [4]Division of Neurology/Molecular Brain Science, Kobe University Graduate School of Medicine, 7-5-1 Kusunoki-chou Chuo-ku, Kobe 650-0017, Japan; [5]Institute of Biological Chemistry, Academia Sinica, 128, Academia Road Sec 2, Nankang, Taipei 115, Taiwan; [6]Okazaki Institute for Integrative Bioscience and Institute for Molecular Science, National Institutes of Natural Sciences, 5-1 Higashiyama Myodaiji, Okazaki 444-8787, Japan.

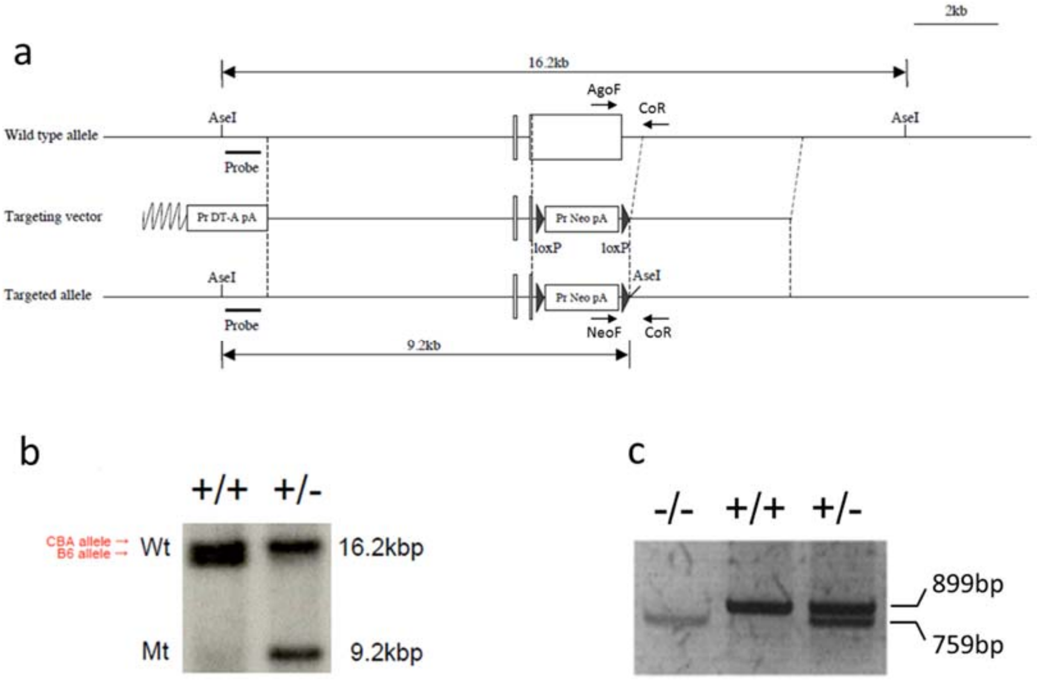

Fig. S1 | Targeted distribution of the AGO61 gene. (a) Schematic representation of the wild-type gene, a predicted mutant allele, and the targeting vector. (b) Southern blot analyses of genotypes using mouse tail tissues. Endogenous (16.2 kbp) and targeted (9.2 kbp) AGO61 alleles. (c) PCR genotyping of embryos at embryonic day 13.5. An AgoF and CoR primer set was used to identify the endogenous allele (899 bp), and a NeoF and CoR primer set was used for the targeted allele (759 bp).

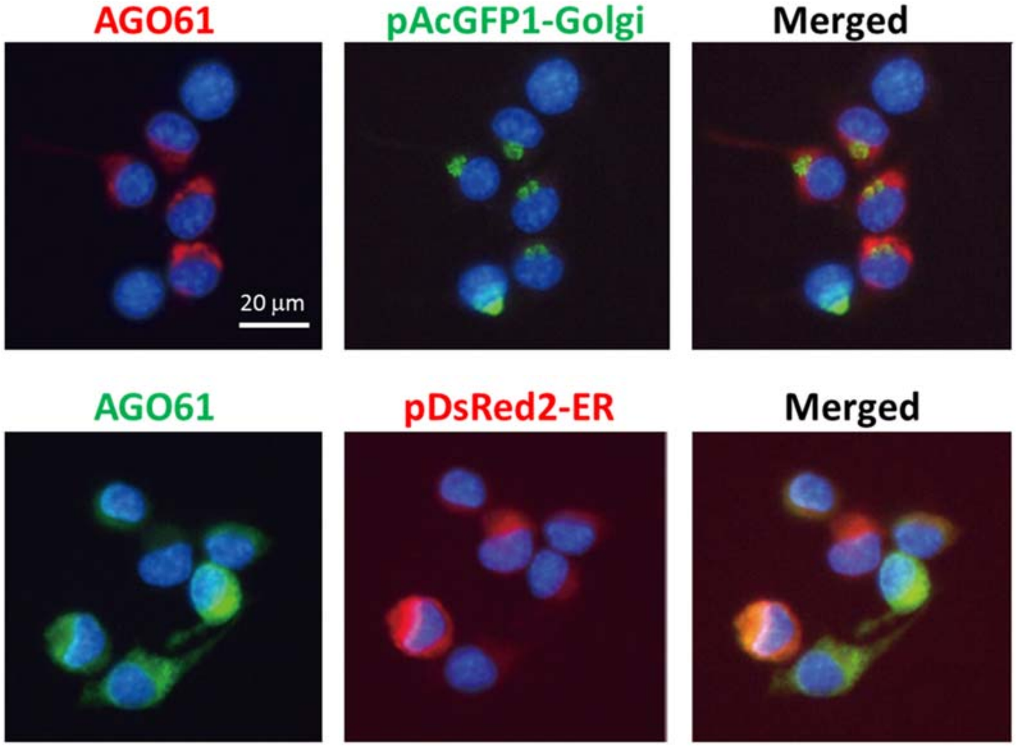

Fig. S2 | AGO61 is localized at the endoplasmic reticulum. AGO61 was transiently transfected with GFP1-Golgi or DsRed2-ER into Neuro2a cells. Neuro2a cells were treated with PBS containing 3% fetal bovine serum and 0.1% Triton X-100 and then stained with an anti-AGO61 antibody and Alexa Fluor 488-conjugated or 594-conjugated anti-rabbit IgG antibody (*green* or *red*). Nuclei were stained with DAPI (*blue*).

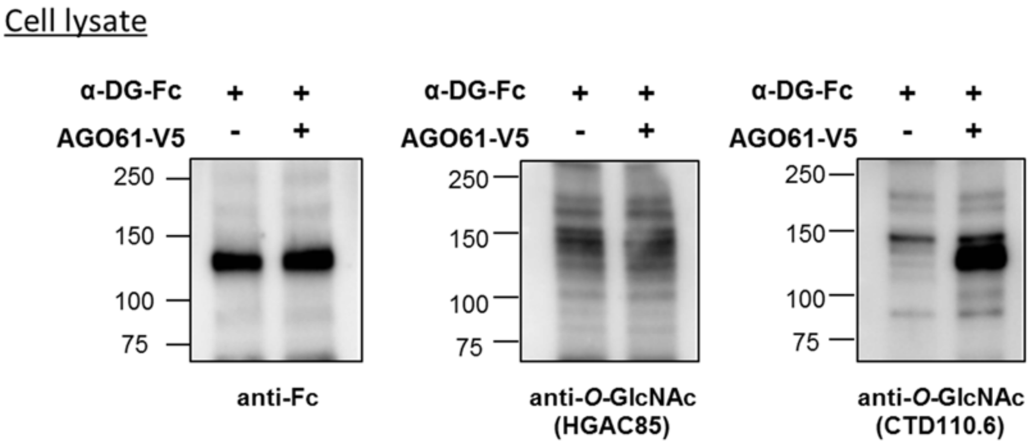

Fig. S3 | The anti-*O*-GlcNAc antibody CTD110.6 recognizes GlcNAc residues on *O*-Man residues as well as *O*-GlcNAc. α-DG-Fc was transiently transfected with or without AGO61 into COS7 cells. Cell lysates were analyzed by Western blot using anti-Fc and anti-*O*-GlcNAc (HGAC85 and CTD110.6) antibodies.

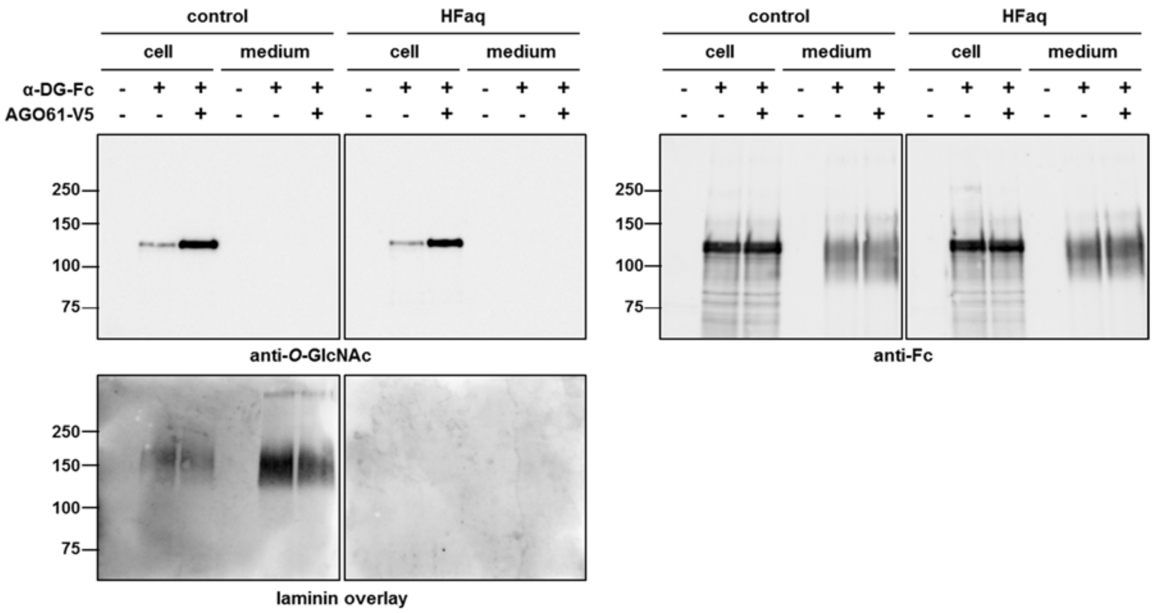

Fig. S4 | A GlcNAc modification remains after HFaq treatment. α-DG-Fc recombinant proteins were collected from cell lysates and culture medium using protein A resin and then analyzed by Western blot using anti-Fc and anti-*O*-GlcNAc antibodies with or without HFaq treatment.

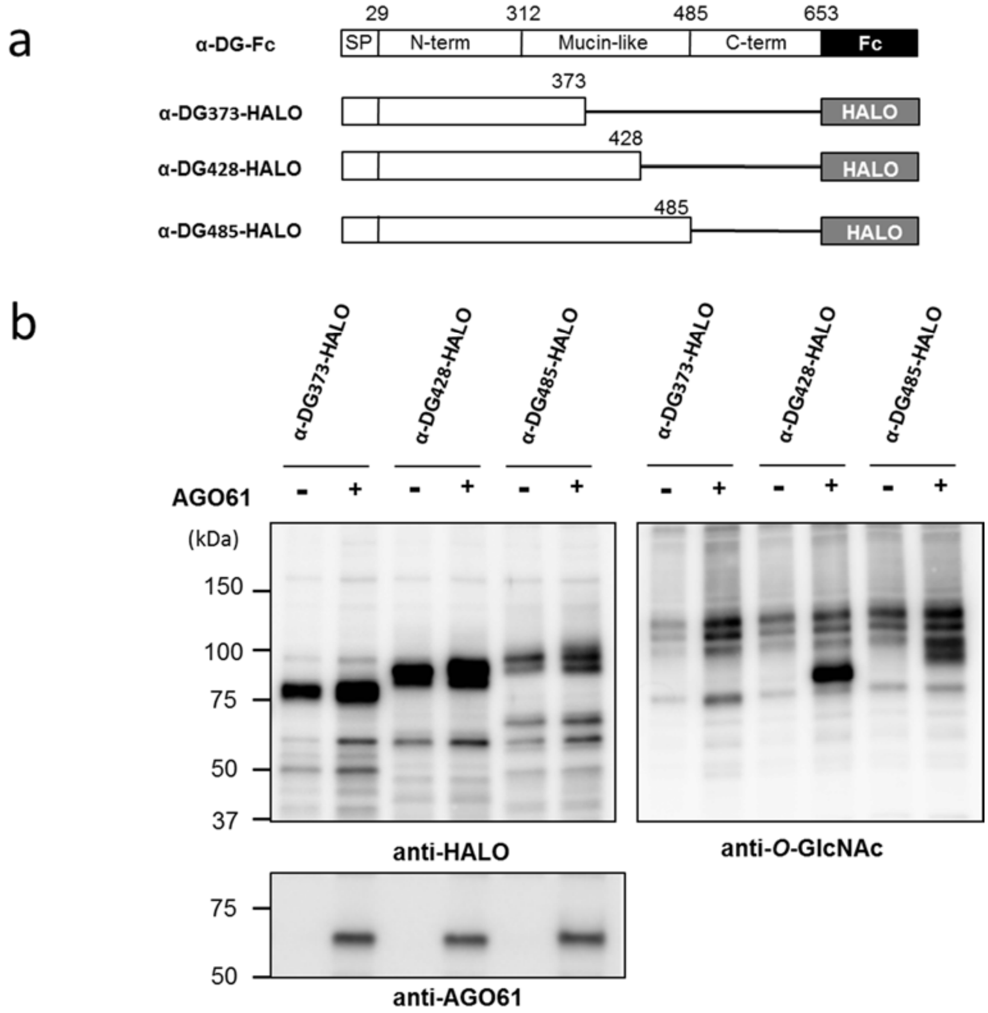

Fig. S5 | AGO61-dependent-GlcNAc modifications on α-DG deletion mutants. (a) Schematic diagrams of α-DG deletion mutants. Mutants comprised a signal peptide (SP), an N-terminal domain (N-term), and a mucin-like domain. (b) α-DG deletion mutants were transiently transfected with or without AGO61 into COS7 cells. Cell lysates were analyzed by Western blot using anti-HALO, anti-AGO61, and anti-*O*-GlcNAc antibodies.

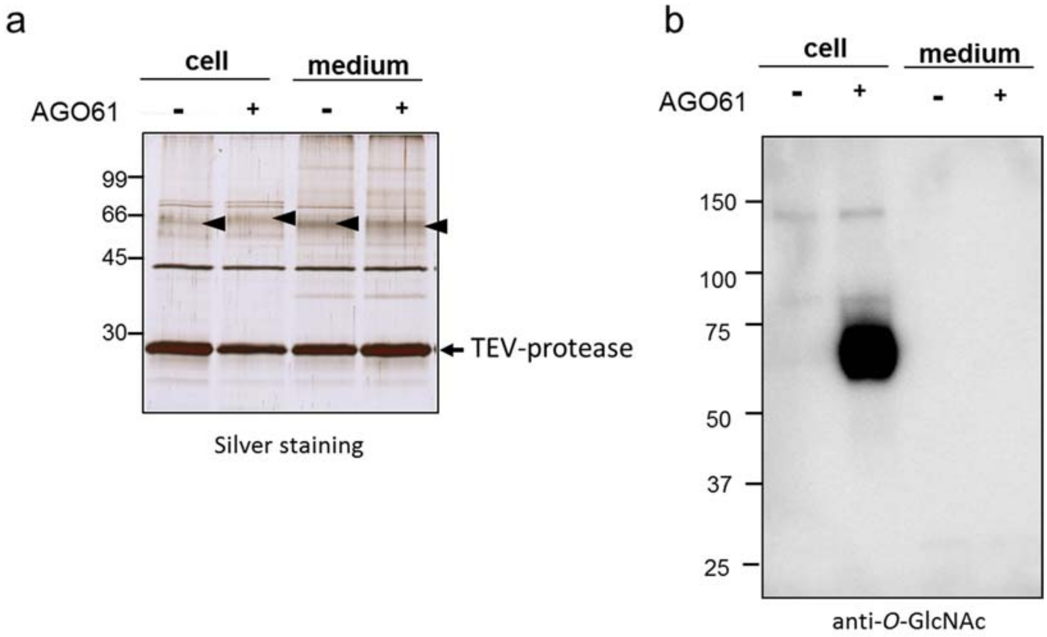

Fig. S6 | Purification of α-DG428 for LC-MS/MS analysis.

α-DG428-HALO was transiently transfected with or without AGO61 into COS cells. (a) α-DG428 was purified from cell lysates and culture media using a HaloTag protein purification system, and then detected by silver staining. Pieces of the destained polyacrylamide gel containing α-DG428 (indicated by arrow) were excised, digested with trypsin, and then subjected to LC-MS/MS analysis. (b) Purified α-DG428 was detected by Western blot using an anti-*O*-GlcNAc antibody.

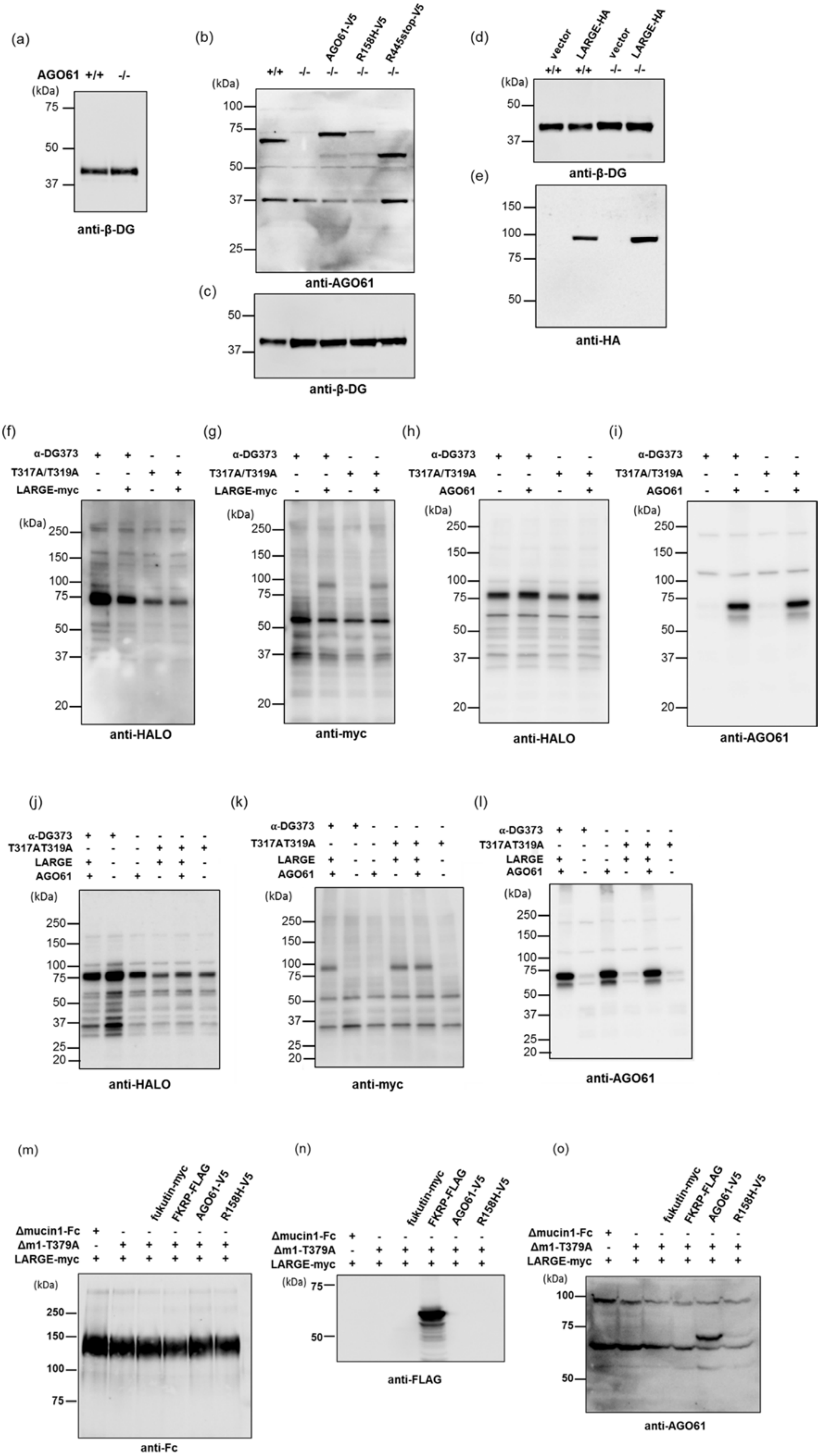
(a)
AGO61 +/+ -/-
(kDa)
75
50
37
anti-β-DG
(b)
AGO61-V5
R158H-V5
R445stop-V5
+/+ -/- -/- -/- -/-
(kDa)
100
75
50
37
25
anti-AGO61
(c)
50
37
anti-β-DG
(d)
vector LARGE-HA vector LARGE-HA
+/+ +/+ -/- -/-
(kDa)
50
37
anti-β-DG
(e)
150
100
75
50
anti-HA
(f)
α-DG373 + + - -
T317A/T319A - - + +
LARGE-myc - + - +
(kDa)
250
150
100
75
50
37
20
anti-HALO
(g)
α-DG373 + + - -
T317A/T319A - - + +
LARGE-myc - + - +
(kDa)
250
150
100
75
50
37
20
anti-myc
(h)
α-DG373 + + - -
T317A/T319A - - + +
AGO61 - + - +
(kDa)
250
150
100
75
50
37
20
anti-HALO
(i)
α-DG373 + + - -
T317A/T319A - - + +
AGO61 - + - +
(kDa)
250
150
100
75
50
37
20
anti-AGO61
(j)
α-DG373 + + - - - -
T317AT319A - - - + + +
LARGE + - - + + -
AGO61 + - + + + -
(kDa)
250
150
100
75
50
37
25
20
anti-HALO
(k)
α-DG373 + + - - - - -
T317AT319A - - - - + + +
LARGE + - - - + + -
AGO61 + - - + + + -
(kDa)
250
150
100
75
50
37
25
20
anti-myc
(l)
α-DG373 + + - - - -
T317AT319A - - - + + +
LARGE + - - + + -
AGO61 + - + - + -
(kDa)
250
150
100
75
50
37
25
20
anti-AGO61
(m)
fukutin-myc FKRP-FLAG AGO61-V5 R158H-V5
Δmucin1-Fc + - - - - -
Δm1-T379A - + + + + +
LARGE-myc + + + + + +
(kDa)
250
150
100
75
anti-Fc
(n)
fukutin-myc FKRP-FLAG AGO61-V5 R158H-V5
Δmucin1-Fc + - - - - -
Δm1-T379A - + + + + +
LARGE-myc + + + + + +
(kDa) 75
50
anti-FLAG
(o)
fukutin-myc FKRP-FLAG AGO61-V5 R158H-V5
Δmucin1-Fc + - - - - -
Δm1-T379A - + + + + +
LARGE-myc + + + + + +
100
75
50
anti-AGO61

Fig. S7 | Full-length blots.

The regions of interest are highlighted in main Figs: (a) Fig.2a; (b and c) Fig.2d; (d and e) Fig.2e; (f and g) Fig.3b; (h and i) Fig.3c; (j, k, and l) Fig.5a; and (m, n, and o) Fig5b.
